# Enhanced intestinal protein fermentation in schizophrenia

**DOI:** 10.1186/s12916-022-02261-z

**Published:** 2022-02-09

**Authors:** Ying Liang, Xing Shi, Yang Shen, Zhuoran Huang, Jian Wang, Changjun Shao, Yanan Chu, Jing Chen, Jun Yu, Yu Kang

**Affiliations:** 1 National Clinical Research Center for Mental Disorders, Peking University Sixth Hospital, Institute of Mental Health, Key Laboratory of Mental Health, Ministry of Health, Peking University, Beijing, China; 2 CAS Key Laboratory of Genome Sciences and Information, Beijing Institute of Genomics, Chinese Academy of Sciences, Beijing, 100101 China; 3 The First Affiliated Hospital (Shenzhen People’s Hospital), Southern University of Science and Technology, Shenzhen, 518055 China; 4 School of Life Sciences, Huaibei Normal University, Huaibei, ,235000 Anhui China; 5 China National Center for Bioinformation, Beijing, 100101 China; 6 University of Chinese Academy of Sciences, No.19 Yuquan Road, Shijingshan District, Beijing, 100049 China

**Keywords:** Schizophrenia, Gut microbiota, Protein fermentation, Macronutrient metabolism, Nutrition care, Dysbiosis, Metagenome, Metabolome

## Abstract

**Background:**

Emerging findings highlighted the associations of mental illness to nutrition and dysbiosis in the intestinal microbiota, but the underlying mechanisms, especially in schizophrenia (SZ), remain unclarified.

**Methods:**

We conducted a case-control study of SZ patients (case to control=100:52) by performing sequencing of the gut metagenome; measurement of fecal and plasma non-targeted metabolome; including short-, medium-, and long-chain fatty acids; and targeted metabolites, along with recorded details of daily intakes of food.

**Results:**

The metagenome analysis uncovered enrichment of asaccharolytic species and reduced abundance of carbohydrate catabolism pathways and enzymes in the gut of SZ patients, but increased abundance of peptidases in contrast to their significantly reduced protein intake. Fecal metabolome analysis identified increased concentrations of many protein catabolism products, including amino acids (AAs), urea, branched short-chain fatty acids, and various nitrogenous derivates of aromatic AAs in SZ patients. Protein synthesis, represented by the abundance of AA-biosynthesis pathways and aminoacyl-tRNA transferases in metagenome, was significantly decreased. The AUCs (area under the curve) of the diagnostic random forest models based on their abundance achieved 85% and 91%, respectively. The fecal levels of AA-fermentative enzymes and products uniformly showed positive correlations with the severity of psychiatric symptoms.

**Conclusions:**

Our findings revealed apparent dysbiosis in the intestinal microbiome of SZ patients, where microbial metabolism is dominated by protein fermentation and shift from carbohydrate fermentation and protein synthesis in healthy conditions. The aberrant macronutrient metabolism by gut microbes highlights the importance of nutrition care and the potential for developing microbiota-targeted therapeutics in SZ.

**Supplementary Information:**

The online version contains supplementary material available at 10.1186/s12916-022-02261-z.

## Background

Schizophrenia (SZ) affects ~1% of the world population and is among the top 10 global causes of disability [1]. The etiology of SZ remains unknown and likely involves a wide range of environmental factors that affect the neurophysiological processes in genetically susceptible individuals [2]. Although some medications of SZ are available and effective in alleviating acute psychiatric symptoms [3], their pharmacological mechanisms remain unclarified, and they are often ineffective in treating negative symptoms. Negative symptoms are a core component of SZ which refer to diminution or absence of normal behaviors related to social activity and motivation and account for the major part of long-term functional outcomes [4]. Due to the largely unknown neurological mechanisms of the negative symptoms, effective medications for them are scarce, leaving the huge medical need remains unmet [2, 5].

Treatment of schizophrenia relies heavily on the use of antipsychotic drugs, which not only regulate the neurotransmitters in the brain, but also impact the metabolism of patients, often leading to weight gain, abnormal blood glucose, dyslipidemia, etc. [6]. The risk of abdominal obesity in patients with chronic schizophrenia is fourfold higher than in the general population, and the risk of diabetes and metabolic syndrome is twofold to threefold higher than in the general population [7, 8]. The weight gain may be related to the activation of some neurotransmitter receptors by antipsychotic drugs, including 5-hydroxytryptamin 2A (5HT2A) and 5-HT2C receptors, and histamine H1 receptor [9]. At the same time, antipsychotic drugs can also affect the expression of leptin gene, reduce leptin sensitivity, and induce weight gain.

Recent studies have shown the presence of chronic, low-level elevated immune markers in schizophrenia [10, 11]. The cytokines that change in schizophrenia have similar changes in metabolic disorders, such as interleukin-1Rα(IL-1Rα), IL-1β, IL-2, IL-6, IL-10, interferon-γ(IFN-γ), tumor necrosis factor-α(TNF-α), and C-reactive protein (CRP) [12, 13]. Thus, disturbances of the immune system may play an important role in the underlying and drug-induced metabolic disturbances of schizophrenia. In addition, under the influence of psychiatric symptoms, patients with schizophrenia can maintain poor diet and living habits for a long time, which is also one of the important risk factors for patients with abnormal blood glucose and lipids [14]. Therefore, the rehabilitation of patients with schizophrenia needs comprehensive management from many aspects such as antipsychotics, diet, living habits, and so on.

Recently, epidemiological data have highlighted the association between nutrition and mental health, which raises up more studies in the nascent field of nutritional psychiatr y[15]. Although diet has been reported in many studies to potentially prevent or treat mental illness, mostly major depression [16, 17], information about the causality or underlying mechanism is still unclarified. The intestinal microbiota, which has great potentials in metabolizing various nutrients and is in turn regulated by nutrient supply, involved deeply in the interactions between nutrition and mental illness [18]. Multiple routes, such as the vagus nerve, immune system, and endocrine systems, connect the gut microbes to the brain and consequently affect human behaviors [19, 20], forming a nutrients-microbiota-brain axis. In SZ patients, metabolic dysregulation, such as abnormal blood concentrations of amino acids (AAs), has been reported [21, 22], as well as dysbiosis in the intestinal microbiome where a variety of bacterial species were found enriched or absent in patients with this disorder [23–27]. However, the exact mechanisms of how these species affect nutrient metabolism and brain functions are not entirely understood in their complexity. Clinical study that systemically investigates the nutrient intake, gut microbiota, fecal and plasma metabolome, and mental health status in patients of mental illness may help to clarify the associations among nutrients, microbiota, and psychiatric symptoms, as well as the pathophysiology of SZ.

Here, we performed a case-control study of SZ (case: control = 100: 52) where we conducted metagenome shotgun sequencing of stool DNA and analysis of metabolome of non-targeted small molecular metabolites as well as free fatty acids of various chain-length in plasma and fecal samples to dissect the potential roles of nutrients and intestinal microbes in the pathogenesis of SZ. Our results discovered a significantly enhanced intestinal protein fermentation in patients that shifted from carbohydrate fermentation and protein synthesis in healthy conditions. The activity of protein fermentation, represented by relevant enzyme abundance, fermentation product levels, and the daily intake of protein, uniformly exhibited positive correlations to the severity of negative psychiatric symptoms. These findings illustrated a hyperactive fermentative process where an aberrant proportion of ingested proteins were delivered to intestinal microbes and intensively catabolized into AAs and further into nitrogenous products. As the fermentative products of protein are often neurologically active, the enhanced microbial protein fermentation in the gut provides a molecular mechanism for how gut microbes undermine the social behaviors in SZ patients.

## Methods

### Ethical compliance

All experimental protocols were approved by the ethics committee of Gaizhou Renkang Hospital, a sanatorium for patients with mental illness (No. 201801). The ethical conduct of this study met all local legal and regulatory requirements. This study was conducted in accordance with the ethical principles found in the Declaration of Helsinki.

An informed consent form (ICF) explaining the procedures of the study including the potential hazards was reviewed and approved by the ethics committee of Gaizhou Renkang Hospital before its use. ICF was read by and explained to all patients or their legal representative prior to their participation in the study. Each patient had ample opportunity to ask questions, and investigators gave sufficient explanations in a comprehensible language regarding characteristics, objectives, relevant procedure, expected duration of the study, and possible limitations/risks for participating in this study. Also, each patient was assured of their right to withdraw from the study at any time without any disadvantage and without having to provide a reason for their decision. An informed consent was obtained from each patient or legal representative before any study-related procedures were performed. A copy of informed consent was provided to patient with signatures of the patient or legal representative and investigator.

### Subject recruitment

Schizophrenic patients aged 18~65 was recruited from the sanatorium and reassessed with the Mini International Neuropsychiatric Interview (M.I.N.I.) according to the diagnostic criteria of the international classification of diseases (ICD-10). Healthy controls matched in age and sex were recruited from the faculty of the sanatorium or surrounding communities. Subjects in one or more of the following conditions were excluded: (1) was diagnosed with other mental disorders or complicated with other chronic diseases, such as hypertension, diabetes, immune deficiency disease, autoimmune disease, cancer, inflammatory bowel disease, severe diarrhea; (2) had taken antibiotics, probiotics, prebiotics, glucocorticoids, immunosuppressants, and gastrointestinal examination in the past six months; (3) had a history of gastrointestinal or hepatobiliary surgery in recent five years; (4) body mass index (BMI) < 18.5 or > 27.9 kg/m^2^; and (5) in the past three months, subjects had no abnormal eating behavior, such as restrictions on the type or amount of food, pica, vomiting, and diarrhea.

### Clinical information collection

Clinical information was collected for each subject through questionnaires, which included sex, age, medical history, body height, and weight. Information on dietary in the past month was collected for all subjects using the Food Frequency Questionnaire (FFQ) [28], adjusted to 117 items and 15 food types according to Chinese dietary habits. The questionnaire adopts a closed survey method, and each closed question provides nine answer options. Open-ended questions were added at the end of the questionnaire to gather information about other foods not listed. Daily intake of carbohydrates, protein, fat, and other nutrients content was calculated according to the standard amount consumed by food category and the total amount of food in the last month. The severity of psychiatric symptoms was reassessed using the Positive And Negative Symptom Scale (PANSS) [29] for each patient.

### Fecal and plasma samples collection

For each subject, a fresh stool sample was collected in a clean and dry container and immediately transferred to the laboratory in the hospital. Each ~400 mg sample from the central part of the sample was transferred into a sterile tube in triplicate. The tubes were immediately put into liquid nitrogen for storage. Blood samples were collected in 1.5 mL heparin anticoagulant tubes, let stand at room temperature for 1 h, and then centrifuged at 3000 rpm for 10 min. The supernatant was collected and separated in triplicate sterile tubes, then put into liquid nitrogen for storage. All samples were transported in liquid nitrogen and stored at −80°C.

### Metagenome sequencing and annotation

The total DNA of the stool sample was extracted using EZNA® Stool DNA Kit (Omega Bio-Tek Inc.), and the concentration and purity of extracted DNA were assessed by Qubit 2.0 fluorometer (Invitrogen) and NanoDrop 2000 (Thermo Fisher Scientific). Paired-end libraries were constructed using a KAPA HyperPrep Kit (Roche) for Illumina platforms, and paired-end sequencing was performed on Illumina HiSeq X Ten platform (Annoroad, Beijing, China) at 150bp×2 and average 10Gb raw reads for each sample. Quality control, annotation of taxonomy profile and metabolic functions, and reconstruction of quasi-paired cohort were performed routinely or as previously described [30], refer to Additional file 1: Supplementary Methods for details.

### Metabolome analysis of fecal and plasma samples

The analysis of short-chain fatty acids, medium- and long-chain fatty acids, and non-targeted metabolome were performed following routine operations by Tinygene Bio-Tech (shanghai) Co., Ltd. For each subject, three aliquots of 100 μl plasma were respectively applied to the analysis of short-chain fatty acids, medium- and long-chain fatty acids, and non-targeted metabolome, and fecal samples (~400 mg) went for the same analyses after pretreatment of grinding and sonication. Refer to Additional file 1: Supplementary Methods for details. For targeted fecal metabolomic detection, internal standard references of amino acids and related metabolites were purchased from Cambridge Isotope Laboratories (U.S), and the methods of metabolite extraction, instrument, and data analyzing of target metabolomic detection were conducted as previously described [31] with modification, refer to Additional file 1: Supplementary Methods for details.

### Statistical analysis

SPSS 19.0 was used to analyze clinical and dietary data. The results were expressed as mean ± standard deviation or percentage. The *t*-test (double-tailed) and the Wilcoxon rank-sum test were used to determine significant differences between the two groups, and the chi-square test was used to compare the differences between the two groups for classification variables. *P*-value < 0.05 and FDR < 0.1 was considered to be significant.

The significance of the difference in nutrient intake, carbohydrate-Active enzymes (CAZys), peptidases, species, short-chain fatty acids (SCFAs), and metabolites were calculated based on the Wilcoxon rank-sum test. Differential pathways between quasi-paired cohorts were performed using the Wilcoxon signed-rank test for paired samples. Correlations of species, enzymes, metabolites, and symptom scores were conducted using Spearman’s rho. Features selection of metabolites and species was performed using Boruta package (https://cran.r-project.org/web/packages/Boruta/) with the default parameters, in which a shuffled data matrix was built, and Random Forest classifier was used to compute the importance score for each feature in the original data and the shuffled data, and features were reported as important by comparing the importance scores (100 iterations, *p* < 0.01).

The abundance of 34 AA-biosynthesis pathways or 22 aminoacyl-tRNA transferases were used to construct random forest classifiers with the packages of caret [https://cran.r-project.org/web/packages/caret/] and randomForest [https://cran.r-project.org/web/packages/randomForest/] in R. The model was trained with 2/3 of all samples and tested by the other 1/3 samples with 1000 times of bootstrapping. The performance of the model was evaluated by the Receiver operating characteristic curve (ROC) analysis with Scikit-learn v0.21.2 (https://scikit-learn.org/stable/whats_new/v0.21.html#version-0-21-2), and ROC curve was plotted using matplotlib 3.0.3. Curve fitting and statistical calculation were performed using ggplot2 (https://ggplot2.tidyverse.org) package. All these data procession and calculation were performed using R.

## Results

### Demographic data

A total of 116 patients with chronic schizophrenia were enrolled, and 100 patients eventually met the criteria. The age, sex ratio, and course of disease in patients’ group were 43.11±9.81 years, 34: 66 (F/M), and 13.35±8.53y ears. In all patients, the levels of fasting blood glucose, triglyceride, and total cholesterol were within normal range(4.3±0.7mmol/L, 1.1±0.8mmol/L, 3.8±0.7mmol/L). There was no statistical difference between the patients and healthy controls. All patients were treated with antipsychotic medications, the equivalent dose of chlorpromazine was 246.0±179.5 mg/day. In these cases, ten patients (10.0%) were treated with polypharmacy of two antipsychotics, and others (90.0%) were treated with monotherapy. Ninety-four patients received atypical antipsychotic drugs (Risperidone =51, dose 3.8±1.4mg/day; Olanzapine =5, dose 10.0±0.0mg/day; Quetiapine =9, dose 388.9±183.3mg/day; Aripiprazole =6, dose 11.7±5.2mg/day; Clozapine =19, dose 205.3±100.5mg/day; Amisulpride =4, dose 562.5±540.6mg/day), and 20 patients received typical antipsychotic drugs (Haloperidol =2, dose 6.0±2.8mg/day; Chlorpromazine =8, dose 268.8±196.3mg/day; Perphenazine =10, dose 18.0±14.8mg/day).

### Unbalanced nutrition and altered metabolome in patients with schizophrenia

During May ~ Aug 2018, the study enrolled 100 SZ patients from a sanatorium for patients of mental illness in County Gai, Liaoning, China, and 52 age- and sex-matched healthy controls who were staff members of the sanatorium or inhabitants of the same town in order to minimize the influence of food sources and dietary habits in gut microbiota [32]. Demographic and clinical information, and dietary records of the recent month before enrollment, were collected in detail for each subject (Table 1). Stool and plasma samples of all subjects were collected upon informative consent and cryopreserved for subsequent shotgun sequencing and metabolome analysis.

**Table 1:** Demographic, clinical characteristics, and dietary patterns of all subjects

Variable	Schizophrenia (***n***=100)	healthy controls (***n***=53)	***p*** value
**Demographic characteristics**			
Age, means (SD)	43.11 (9.81)	48.92 (12.95)	0.005
Education year, means (SD)	7.80 (2.79)	9.34 (3.29)	0.003
Height, means (SD)	1.64 (0.07)	1.64 (0.08)	0.645
weight, means (SD)	60.53 (7.86)	65.04 (8.20)	0.001
BMI, means (SD)	22.59 (2.44)	24.11 (2.45)	<0.001
Female, No. (%)	34 (34)	21 (39.6)	0.490
Married, No. (%)	39 (39)	48 (90.6)	<0.001
Smoking, No. (%)	0	11 (20.8)	<0.001
Drinking, No. (%)	0	10 (18.9)	<0.001
**Clinical characteristics**			
Fasting plasma glucose, mmol/L, means (SD)	4.26 (0.65)	4.40 (0.35)	0.252
Triglycerides, mmol/L, means (SD)	1.06 (0.83)	1.07 (0.33)	0.888
Cholesterol, mmol/L, means (SD)	3.80 (0.74)	3.77 (0.85)	0.844
Course of disease, years, means (SD)	13.35 (8.53)	NA	NA
Monotherapy , No. (%)	81 (81)	NA	NA
Equivalent dose of chlorpromazine, means (SD)	246.00 (179.51)	NA	NA
Positive syndrome score, means (SD)	17.45 (5.56)	NA	NA
Negative syndrome score, means (SD)	24.36 (7.26)	NA	NA
General syndrome score, means (SD)	39.76 (5.88)	NA	NA
PANSS total score, means (SD)	81.51 (10.95)	NA	NA
**Dietary characteristics, means (SD)**			
Energy, kcal/day	1595.03 (106.97)	1817.25 (295.00)	<0.001
Water, g/day	982.15 (32.53)	996.84 (122.75)	0.396
Protein, g/day	58.15 (8.16)	97.57 (19.67)	<0.001
Fat, g/day	82.31 (8.52)	83.95 (26.04)	0.658
Carbohydrate, g/day	155.27 (9.74)	167.76 (20.86)	<0.001
P%	14.59 (1.9)	21.42 (2.6)	<0.001
F%	20.6 (0.9)	18.18 (2.7)	<0.001
C%	39.0 (2.2)	37.6 (6.8)	0.16
Dietary fiber, g	12.84 (1.61)	16.37 (2.65)	<0.001
Retinol equivalent, μg	788.98 (84.04)	841.69 (143.98)	0.017
Vitamin B1, mg	0.78 (0.06)	1.13 (0.27)	<0.001
Vitamin B2, mg	0.96 (0.05)	1.49 (0.42)	<0.001
Vitamin PP, mg	20.46 (2.98)	21.32 (3.98)	0.173
Vitamin E, mg	18.56 (3.95)	26.87 (8.62)	<0.001
Na, mg	1260.04 (188.79)	2118.78 (449.25)	<0.001
Ca, mg	432.72 (31.07)	543.71 (134.25)	<0.001
Fe, mg	17.75 (5.22)	25.58 (5.32)	<0.001
Vitamin C, mg	119.39 (5.48)	114.46 (8.46)	<0.001
Cholesterol, mg	471.40 (39.49)	805.65 (277.21)	<0.001

**Fig. 1:**
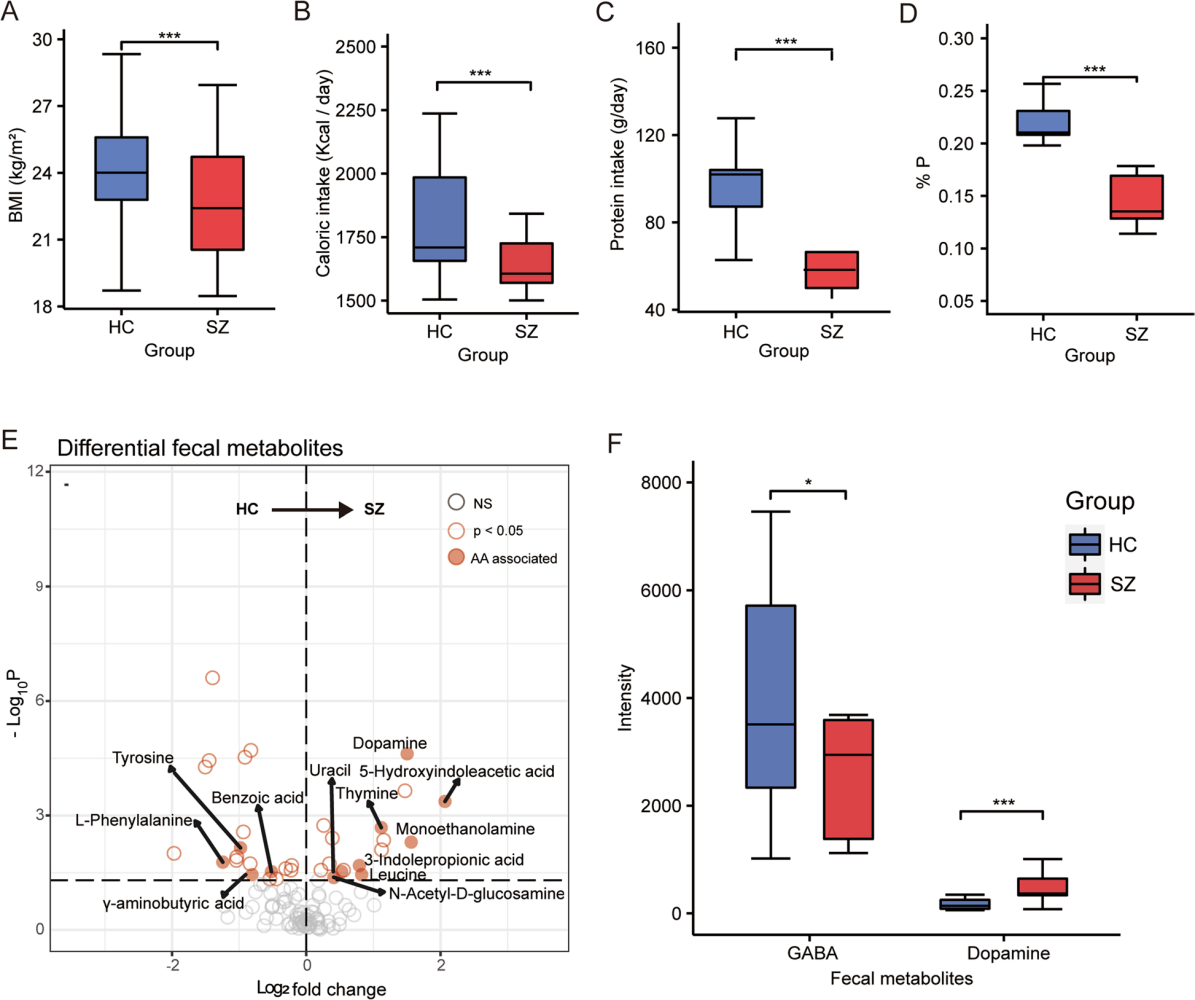
Unbalanced nutrition and dysregulated intestinal metabolism in SZ patients. **A–D** Differences in BMI (**A**), daily calorie intake (**B**), protein intake (**C**), and the percentage of calories provided by ingested protein (%P) (**D**) between HC and SZ groups. **E** Volcano plot of the differential metabolites in stool. **F** Fecal concentrations of GABA and dopamine between HC and SZ groups. HC, healthy controls; SZ, patients with schizophrenia; GABA, γ-aminobutyric acid. *p < 0.05, ***p < 0.001, Wilcoxon rank-sum test

We first noticed apparent underweight in SZ patients and a significantly reduced average BMI despite adequate food supply for all patients (Fig. 1A), which observation was in concordance with previous reports from other countries [33, 34]. The daily total calorie intake calculated from the dietary records in SZ patients was significantly reduced (Fig 1B), mainly due to the almost halved protein intake (Fig. 1C) and proportion of calories provided by ingested protein (%P, Fig. 1D). This result suggested a general unbalanced nutrition status in SZ patients, especially the inadequate ingestion of food proteins.

Non-targeted metabolomics analysis for plasma and fecal samples were first performed to investigate the influence of the aberrant macronutrients on metabolism. Comparison of the plasma metabolism between cases and controls identified little differences related to protein catabolism, and among the 14 metabolites significantly altered (Wilcoxon rank-sum test, *p* < 0.05 and FDR < 0.1, Additional file 2: Fig. S1A), only urea and α-hydroxyisobutyric acid (elevated in control samples) were products of protein catabolism. In contrast, the fecal metabolome revealed more extensively altered protein metabolism in SZ patients, and 12 of the 35 significantly altered metabolites (Wilcoxon rank-sum test, *p* < 0.05 and FDR < 0.1 Fig. 1E) were products or related derivates of protein catabolism. The most notable alterations were the elevated levels of the neuroactive dopamine and decrease in the neuroleptic γ-aminobutyric acid in SZ patients (Fig. 1F), both of which are AA derivates and important neurotransmitters in the pathogenesis of SZ.

We then performed metabolome-wide association studies (MWASs) using random forest-based machine learning variable selection techniques to identify fecal metabolite features that deviated SZ. The permutation importance of each metabolite showed that three out of the eight top-ranking metabolites favoring SZ were AA derivates, whereas none metabolites favoring control related to protein catabolism (Additional file 2: Fig. S1B). The apparent differences in fecal metabolism suggested potentially dysregulated protein metabolism in SZ patients, possibly the results of both aberrant protein ingestion and the intestinal microbiome dysbiosis.

To test the alteration in fatty acid metabolism in previous observation [35], we performed absolute quantification of the plasma and fecal levels of medium- and long-chain free fatty acids using gas chromatography-mass spectrometry (GC-MS). Our results confirmed significant deficiency in the plasma levels of some free fatty acids in SZ patients (Wilcoxon rank-sum test, *p* < 0.05 and FDR < 0.1 Additional file 2: Fig. S2). However, the deficiency seemed not to stem from the intestinal metabolism as no difference in fatty acids was identified in the stool (Wilcoxon rank-sum test, *p* < 0.05 and FDR < 0.1 Additional file 2: Fig. S2), given the fact that fats from food are primarily absorbed in the jejunum with little proportion delivered to the intestinal microbiota. Thus, the altered fatty acid profile in plasma is less likely associated with the intestinal microbiome dysbiosis.

### Shifted gut microbial fermentation from carbohydrates to proteins in schizophrenia

**Fig. 2:**
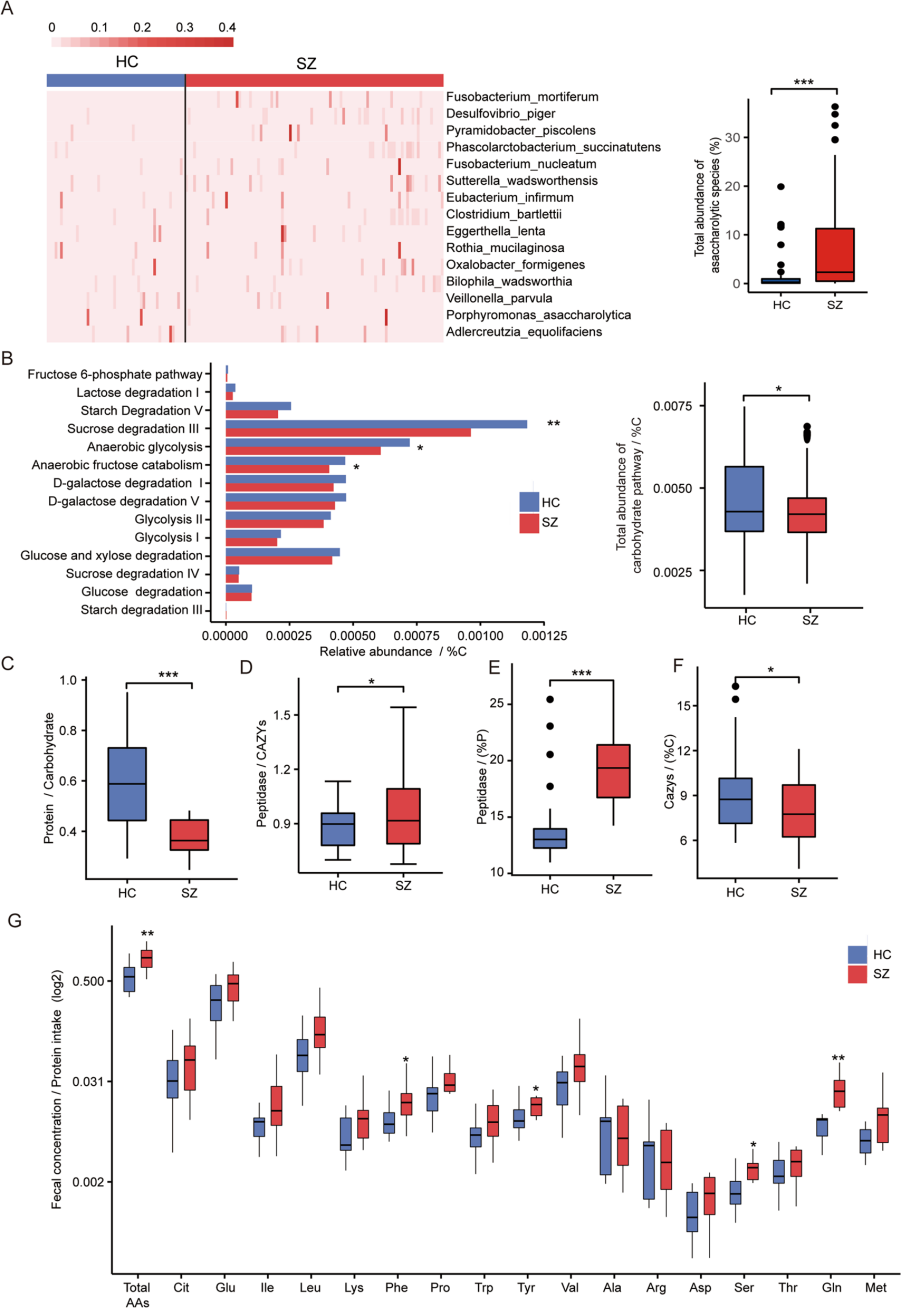
Intestinal metabolism shift from carbohydrate fermentation to proteolysis in SZ patients. **A** The abundance of each asaccharolytic species (left panel) and their sum (right panel) in HC and SZ samples. **B** The relative abundance of each carbohydrate catabolism pathways (left panel) and their sum (right panel) in HC and SZ samples normalized by %P. **C** The ratio of protein to carbohydrate intake in HC than SZ subjects. **D** The ratio of peptidase to CAZys abundance in HC and SZ samples. **E**, **F** The total abundance of peptidases normalized by %P (**E**) and CAZys normalized by %C (**F**) in HC and SZ samples. **G** The fecal concentration of each amino acids and their sum (boxes on the far left) in HC and SZ samples normalized by daily protein intake. HC, healthy controls; SZ, patients with schizophrenia; *p < 0.05; **p < 0.01; ***p < 0.001, Wilcoxon rank-sum test

To investigate how gut microbes participate in the metabolic disturbance in SZ, we performed shotgun-sequencing of the metagenome for all participants. General analysis of the alpha and beta-diversity of the microbiota resulted no significant difference between patients and controls even separately compared in sexes (Additional file 2: Fig. S3). In the analysis of differentially represented species (Wilcoxon rank-sum test, *p* < 0.05 and FDR < 0.15, Additional file 3: Table. S1), we noticed that 4/22 of the species enriched in patients with highest significance and fold-change were asaccharolytic species, i.e., microbes that are unable to metabolize carbohydrates and must rely on other carbon sources for energy, including *Fusobacterium mortiferum*, *Desulfovibrio piger*, *Phascolarctobacterium succinatutens*, and *Sutterella wadsworthensis*, whereas no asaccharolytic species enriched in controls. In the microbiome-wide association studies (MWASs), using random forest model to select taxonomic features of SZ and the top three species of highest permutation importance in favor of SZ was all asaccharolytic (Additional file 2: Fig. S4A). In contrast, no asaccharolytic species were in the list of top permutation importance in favor of controls (Additional file 2: Fig. S4A). Furthermore, we combed all asaccharolytic species represented in our samples and found that most of them, especially those of higher abundance, were enriched in SZ patients, leading to the total abundance of asaccharolytic species significantly elevated in patients (Fig. 2A).

Comparison in metabolic pathways between cases and controls discovered that the total abundance of carbohydrate catabolism pathways, as well as two major ones, i.e., starch degradation and anaerobic glycolysis, were significantly reduced in patients (Additional file 2: Fig. S4B). The reduction, when normalized by the proportion of calories provided by ingested carbohydrates in daily calorie intake (%C), was more remarkable (Fig. 2B), indicating the carbohydrate catabolism was hypoactive in SZ. The hypoactivity in microbial carbohydrate metabolism, together with the enrichment of asaccharolytic species in patients, implies reduced carbohydrate supply to intestinal microbes and hypoactive carbohydrate fermentation by them in the intestine of SZ patients.

Further, according to the MEROPS (database of proteolytic enzymes) and CAZymes (carbohydrate-active enzymes database), we annotated all genes of peptidases and CAZys, which account for the hydrolyzation of protein and carbohydrates into monomers. The initial comparison of their abundance between patients and controls resulted in no significant difference. However, when considering the great difference between the two groups in the ratio of protein to carbohydrate intake (Fig. 2C), the ratio between peptidase and CAZys was significantly reversed (Fig. 2D). Additionally, when normalized by the proportion of calories provided by ingested protein (%P) or carbohydrate (%C) in daily caloric intake, the abundance of peptidases was significantly increased in SZ (Fig. 2E) and reduced in CAZy (Fig. 2F).

To test whether more proteins were hydrolyzed in patients’ gut, we performed targeted chromatographic assay to quantify the concentrations of all AAs in the stool. The result exhibited that most AAs’ fecal concentrations increased in patients, and the total AA concentration was significantly higher than controls, which were more significant when normalized by daily protein intake (Fig. 2G). Notably, we observed significantly elevated fecal concentrations of phenylalanine, tyrosine, and glutamine, which are neurotoxic at high levels in the brain [36] and neural-effective on enteric nerves [37, 38]. All the above observations suggested that, in SZ patients, more undigested proteins reach the colon and are hydrolyzed by microbes there, instead of mostly being hydrolyzed and absorbed in the small intestine in normal conditions.

### Enhanced microbial amino acid catabolism instead of protein biosynthesis in schizophrenia

**Fig. 3:**
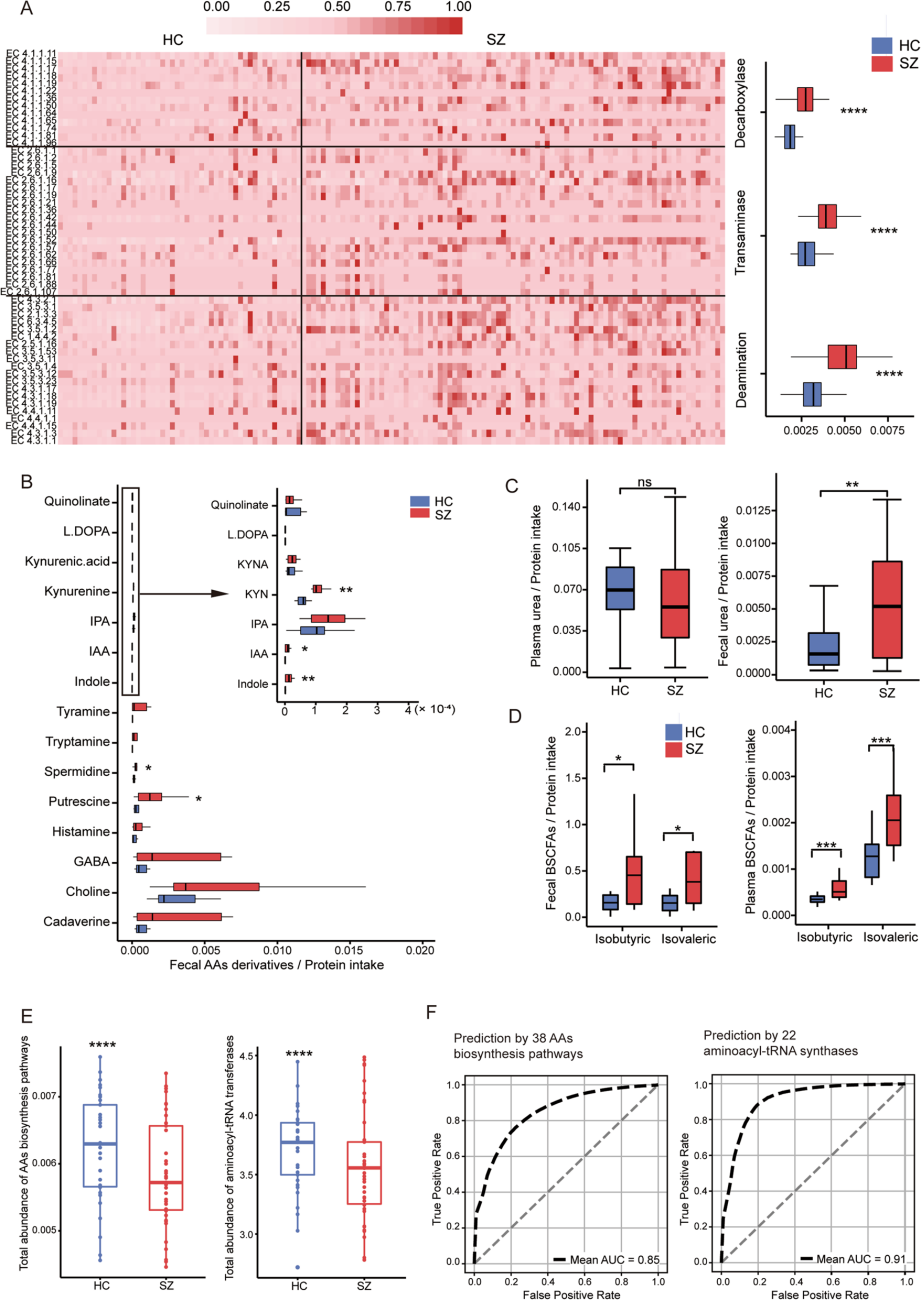
Enhanced amino acids catabolism in SZ patients. **A** The relative abundance of each enzyme accounting for amino acid decarboxylation, transamination, and deamination (left panel) and the sum abundance of each category (right panel) normalization by %P in SZ and HC samples. The color in the heatmap is normalized by Z-score. **B** The fecal concentrations of amino acid derivatives normalized by daily protein intake in SZ and HC samples. **C**, **D** Plasma and fecal concentrations of urea (**C**) and BSCFAs (**D**) normalized by daily protein intake in HC and SZ samples. **E** The total abundance of amino acid biosynthesis pathways (left panel) and aminoacyl-tRNA synthases (right panel) in HC and SZ samples. ****p < 0.0001, Wilcoxon signed-rank test. **F** ROC curve of the diagnostic models based on the abundance of amino acid biosynthesis pathways (left panel) and aminoacyl-tRNA synthases (right panel). KYN, kynurenine; IAA, indole-3-acetic acid; IPA, indole-3-propionic acid; GABA, γ-aminobutyric acid; BSCFA, branched short-chain fatty acid. HC, healthy controls; SZ, patients with schizophrenia; *p < 0.05; **p < 0.01; ***p < 0.001, Wilcoxon rank-sum test

Microbes are powerful in catabolizing AAs into a great variety of derivatives [39, 40], many of which, including amines, nitric oxide (NO), indole, kynurenine, and quinolone, are neurologically active or affect human behavior through actions on the immune or endocrine system [41, 42]. Key enzymes of AA-catabolism fall into three categories that participate in the decarboxylation, transamination, and deamination of AAs, respectively. We then compared their abundance and found that SZ patients harbored more enzymes in all three categories, and the difference became extremely significant when normalized by their protein intake (Fig. 3A).

Decarboxylases are the major enzymes in the generation of various amines and other derivatives. Using targeted chromatography, we quantitated the major decarboxylation derivatives of AAs in stool and found that all derivatives were elevated in SZ patients, confirming the activated microbial AA decarboxylation (Fig. 3B). Among these derivatives, indole, kynurenine, and IAA (indole-3-acetic acid), all derived from tryptophan, showed a significant increase in SZ, which was confirmed by the significantly elevated abundance of enzymes responsible for converting tryptophan into kynurenine and IAA as well (Additional file 2: Fig. S5A). As it has been reported that a variety of tryptophan metabolites are neurologically or immunologically active, the significant increase in tryptophan fermentation products in our study is concordant with some previous reports where tryptophan intake was associated with other brain disorders [41, 43], supporting the potential roles of tryptophan and its metabolites in the pathogenesis of SZ.

Transaminases and deaminases, encoded by both host and microbes, account for catabolizing AAs into ammonium and urea. The urea concentration, when quantitatively measured and normalized by protein intake, showed significantly elevated in stool in SZ patients but no difference in plasma, which indicated activated microbial production of urea in SZ (Fig. 3C). Microbial fermentation is the primary source of SCFAs, including branched short-chain fatty acids (BSCFAs) and straight-chain SCFAs. BSCFAs, including isobutyric and isovaleric acid, are only generated from transamination of branched-chain AAs, i.e., Ile, Leu, and Val [44], while straight-chain SCFAs derived from both AAs (i.e., lysine) and carbohydrates (i.e., dietary fibers) [39]. We then performed targeted chromatographic analysis to quantify the concentration of all SCFAs in both plasma and stool. The results showed that both fecal and plasma concentrations of BSCFAs significantly increased in patients (Fig. 3D). In contrast, no significant differences were detected in both plasma and fecal concentrations of SCFAs (Additional file 2: Fig. S5B), supporting the enhanced AA-catabolism in the gut of SZ patients.

To define the deviations in the metabolic profiles between SZ and controls, we utilized a recently developed method for metagenome analysis-Quasi-paired cohort [30]. In the list of differential metabolic pathways identified by the Quasi-paired cohort (Wilcoxon signed-rank test for paired samples, FDR < 0.001, mean abundance in control > 10^-5^, Additional file 3: Table. S2), among pathways enriched in controls, 8/31 (26%) were in the category of AA-biosynthesis, whereas none in this category overrepresented in SZ. Notably, we also compared the abundance of aminoacyl-tRNA synthases (EC 6.1.1) between the paired SZ-control samples, and 12 out of the 22 synthases were significantly enriched in controls (Wilcoxon signed-rank test for paired samples, FDR < 0.05) with the other ten enzymes showed no difference in-between. Comparing the total abundance of the 38 AA-biosynthesis pathways and the 22 aminoacyl-tRNA transferases revealed extremely significant deficiency in the functional potential of protein synthesis in SZ patients (Fig. 3E).

We further constructed random forest classifiers based on the abundance of the 38 AA-biosynthesis pathways and the 22 aminoacyl-tRNA transferases, respectively, and the models performed excellently in discriminating cases and controls and achieved AUC (area under the curve) of 0.85 and 0.91, respectively, when evaluated with ROC (receiver operating characteristic) curve (Fig. 3F). This result indicated that a shift from protein synthesis in normal conditions to protein catabolism was a major deviation in the intestinal metabolism in SZ patients.

### Associations between gut protein fermentation and the impairment of social behaviors

Finally, we investigated whether the protein fermentation in SZ patients correlated to the severity of their psychiatric symptoms, which was reassessed by the PANSS [29] for all patients. For each patient, the total score of negative symptoms (N), positive symptoms (P), general symptoms (G), and total score of all symptoms (T) were calculated and used as indicators for the severity of the disease.

**Fig. 4:**
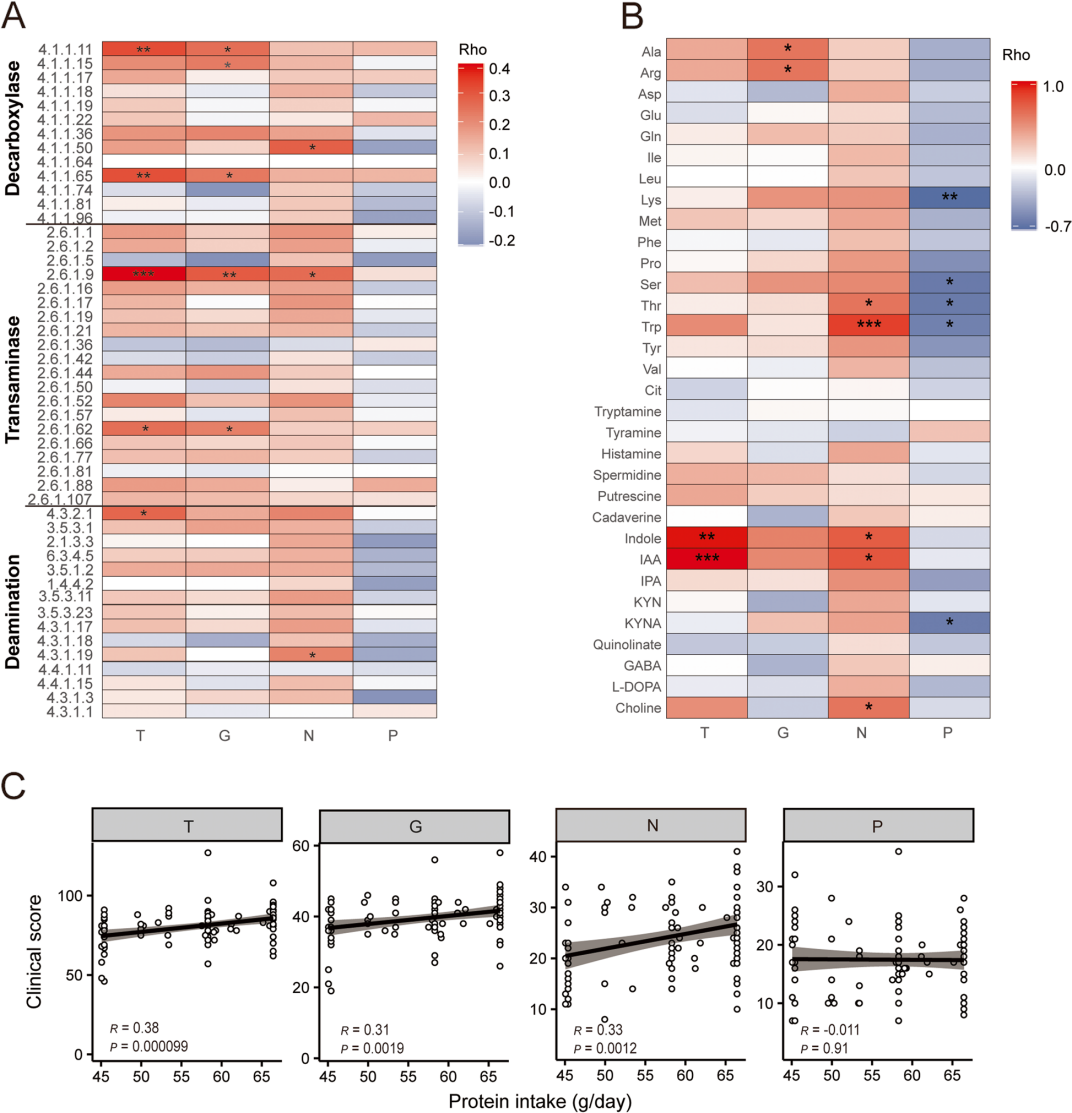
The correlation between microbial protein fermentation and the severity of psychiatric symptoms. **A** The correlations between the relative abundance of amino acids catabolizing enzymes and the clinical score of total score of psychiatric symptoms (T), score of general symptoms (G), negative symptoms (N), and positive symptoms (P). **B** The correlation between the fecal concentrations of amino acids and their derivatives and each of the clinical scores of psychiatric symptoms. The color bar indicates the rho value of Spearman’s rank correlation coefficient; red, positive correlation; blue, negative correlation. **C** The correlation of daily protein intake with the clinical scores of psychiatric symptoms. R Pearson correlation coefficient

First, we investigated the correlations of AA-catabolizing enzymes to clinical scores and observed that these enzymes exhibited correlations positive to N, but negative to P. Although the correlations were not strong, they were uniform among all the enzymes (Fig. 4A). Supportively, the fecal concentration of AAs and their derivatives also presented similar correlations to clinical scores (Fig. 4B). The irrelevance of positive symptoms to intestinal protein fermentation is not surprising as many cofactors such as apastia (food refusal) and psychiatric medications might alleviate positive symptoms [45] and conceal the exacerbating effects of protein fermentation on them. These results suggested a potential association of intestinal microbial protein fermentation to the severity of psychiatric symptoms, especially the negative ones, which representing the unsolved central pathogenesis of SZ.

In concordance with the hypothesis that the enhanced intestinal protein fermentation might impair human behaviors, we even observed positive correlations of daily protein intake to N, G, and T, but not to P (Fig. 4C). In contrast, the daily carbohydrate intake showed no significant correlation to any psychiatric symptoms (Additional file 2: Fig. S6). It seems the food supply of proteins but not carbohydrates associated with severe symptoms, highlighting the role of macronutrient intake in the pathogenesis of SZ. In this point of view, the spontaneous reduction in the daily protein intake in SZ patients might be a protection against harmful protein fermentation products from the gut.

As antipsychotic medications have been reported to influence metabolism, we further investigated their effects on the microbial metabolism of macro-nutrients. Patients were first grouped according to their equivalent dose of chlorpromazine, and there were no significant differences between groups of high and low-dose in their microbial composition and metabolism of protein or carbohydrates. Additionally, we did not observed correlations between the dose of antipsychotic medications and microbial metabolism of macro-nutrition.

## Discussion

Our findings uncovered an aberrant microbial metabolism in the gut of SZ patients characterized as a shift from carbohydrate fermentation to protein fermentation. Although the shift is also reported in other ill-conditions, such as constipation [46] and colon-rectal cancer [47], the deviation towards protein fermentation in SZ seems much more intricate considering the significantly reduced protein intakes. The correlations between proteolysis and the severity of symptoms in our study imply its potential role in the pathogenesis of schizophrenia. Various metabolites generated from protein fermentation have been reported to be active in regulating neural, immune, or endocrine activities, and they often have detrimental effects on brain disorders via the gut-brain axis [41, 48]. It is conceivable that the altered milieu of protein fermentation products in the intestinal lumen may send abnormal signals to the brai n[49], which results in abnormalities in behaviors and arouse psychiatric symptoms in genetically susceptible individuals.

Previous studies have reported altered serum concentration of AAs [21, 22], fatty acids [35], and other metabolites [50] in SZ. These findings, together with the usual observations of moderate underweight and abnormal appetite in SZ patients [33, 34], point to a metabolic disturbance in SZ [51]. As a primary contributor to nutrient metabolism, the intestinal microbiome has been found altered in SZ patients in some cohort studies, where many microbial species were reported enriched or deficient in patients [52]. However, these studies, primarily based on 16S amplicon sequencing, are not always concordant in the discovered species due to the high variability and diversity of the gut microbiome, nor the differential microbial species show evident coherence in their metabolic potentials [24, 53, 54]. The only previously published study based on whole metagenome sequencing (WMS) also reported altered abundance in some microbial species and metabolic pathways in SZ, especially enhanced tryptophan metabolism correlated to clinical manifestation [25]. These findings implied a potential role of intestinal proteolysis in schizophrenia.

Our study, which is based on multi-omics data and in the context of dietary nutrients, thoroughly explored the metabolic alterations of SZ patients using a well-controlled cohort. All our results point to a saliently enhanced gut microbial protein fermentation and the reduced carbohydrate fermentation and protein biosynthesis in SZ. The supportive evidence includes enriched asaccharolytic species, peptidases, and AA-catabolizing enzymes, as well as the reduced abundance of carbohydrate degrading pathways and enzymes, AA-biosynthesis pathways, and aminoacyl-tRNA synthases for protein biosynthesis in metagenomic data; increased fecal concentrations of AAs and their derivates, including urea, BSCFAs, and other neuroactive or immune-active nitrogenous metabolites in metabolome data. The association of enhanced protein fermentation in the pathogenesis of SZ is further corroborated by the diagnostic values of the fecal concentrations of AA derivates and abundance of AA-biosynthesis pathways and enzymes for protein biosynthesis in distinguishing SZ patients, as well as uniformed positive correlations of protein fermentative products and enzymes to the clinical scores of psychiatric symptoms. Therefore, the enhanced protein fermentation in the SZ gut microbiome with solid evidence supported by multiple omics data is much more reliable.

Given the enhanced microbial protein fermentation, our study underscores the importance of nutrition care in SZ patients who often exhibit unbalanced nutrition characterized by inadequate protein intake. Therapeutic strategies aiming to rectify the intestinal dysbiosis and callback the deviated gut signals to the brain seem promising [55]. However, nutrition care is still under investigation and attracts inadequate attention in the care of SZ patients [56]. As the digestion and absorption of macronutrients in SZ patients is severely disordered, undigested proteins are excessively supplied for gut microbes, leading to hyperactivated protein fermentation. In this regard, the reduced protein intake seems to moderate the proteolysis-related effects in SZ patients, although unintendedly. Therefore, the nutrition care for SZ patients is fragile and paradoxical: maintain the supply of essential amino acids for the biosynthesis of neurotransmitters in one aspect and avoid excessive delivery of undigested proteins to the gut microbes in the other, a situation similar to that in hepatic encephalopathy. In such situations of psychopathy caused by metabolic disorders, therapeutic strategies aiming to rectify the intestinal dysbiosis and callback the deviated gut signals to the brain seem promising. Relative strategies may include increase carbohydrates that can only be digested by microbes, such as dietary fiber and lactulose, to flourish carbohydrate-fermenting microbes, or supply hydrolyzed proteins or amino acids instead of ordinary proteins to facilitate the absorption in the small intestine and reduce the delivery of undigested proteins to gut protein-fermenting species. However, nutrition care is still under investigation and attracts inadequate attention in the care of SZ patients. Given that abnormally enhanced protein fermentation participates in the pathogenesis of SZ, delicate diet management may improve the long-term prognosis and open a new avenue for the treatment of the disease.

The current study also presents some limitations. Although the sample size of the different cohorts seems appropriate, population-based studies including subjects from different regions and diet habits would be more representative of this condition. In addition, our conclusions are based on the observations of a cross-sectional study, and cohorts of various classes of body mass or nutrition status would be necessary for a better understanding of the strength of our conclusions. Finally, it is an observational study, and the effects of nutrition management on intestinal metabolism and even psychiatric symptoms in a clinical trial would be of great importance for establishing the role of nutrition care in treating this mental disorder.

## Conclusions

Our findings revealed apparent dysbiosis in the intestinal microbiome of SZ patients, where microbial metabolism is dominated by protein fermentation and shift from carbohydrate fermentation and protein synthesis in healthy conditions. The aberrant macronutrient metabolism by gut microbes highlights the importance of nutrition care and the potentials for developing microbiota-targeted therapeutics in SZ.

## Supplementary Information

**Additional file 1.** Supplementary Methods.

**Additional file 2: Figure S1.** Deviated metabolites in plasma and stool samples of SZ. **Figure S2.** Differential medium-and long-chain free fatty acids in plasma and stool between SZ and HC. **Figure S3.** The diversity of gut microbiota in schizophrenia. **Figure S4.** Decreased carbohydrate-catabolizing activity in the intestine of SZ patients. **Figure S5.** Enhanced amino acid-catabolism in the intestine of SZ patients. **Figure S6.** The correlations of daily carbohydrate intake with the psychiatric symptoms.

**Additional file 3: Table S1.** Differential species in the gut microbiota between HC and SZ. **Table S2.** Differential metabolic pathways encoded by gut microbes between HC and SZ.

## Data Availability

The raw metagenome sequencing data reported in this paper have been deposited in the Genome Sequence Archive in BIG Data Center, Beijing Institute of Genomics (BIG), Chinese Academy of Sciences, under accession numbers CRA003445 at http://bigd.big.ac.cn/gsa/s/2gi5Ka06.

## Abbreviations

SZ: Schizophrenia
AAs: Amino acids
AUC: Area under the curve
5-HT: 5-Hydroxytryptamin
IL: Interleukin
IFN: Interferon
TNF: Tumor necrosis factor
ICF: Informed consent form
CRP: C-reactive protein
MINI: The Mini International Neuropsychiatric Interview
ICD-10: The International Statistical Classification of Diseases and Related Health Problems 10th Revision
BMI: Body mass index
FFQ: Food frequency questionnaire
PANSS: Positive and negative symptom scale
SCFAs: Short-chain fatty acids
CAZys: Carbohydrate-active enzymes
GC-MS: Gas chromatography-mass spectrometry
MWASs: Microbiome-wide association studies
MEROPS: Database of proteolytic enzymes
CAZymes: Carbohydrate-active enzyme database
NO: Nitric oxide
IAA: Indole-3-acetic acid
BSCFAs: Branched short-chain fatty acids
N: Negative symptoms
P: Positive symptoms
G: General symptoms
T: Total score of all symptoms
WMS: Whole metagenome sequencing
