# Supplementary material for: Enhanced intestinal protein fermentation in schizophrenia
Source: BMC Med. 2022 Feb 9;20:67. doi: 10.1186/s12916-022-02261-z (PMC8827269; doi:10.1186/s12916-022-02261-z)
Supplement: Supplementary file 1 — Additional file 1. Supplementary Methods. [file 12916_2022_2261_MOESM1_ESM.docx]

**Supplementary Methods**

*Quality control and annotation of metagenome data*

FastQC was used for the quality assessment of sequencing reads. Trimmomatic v0.36 (<http://www.usadellab.org/cms/?page=trimmomatic>) was used for low-quality reads trimming (parameters: SLIDINGWINDOW:4:20 MINLEN:70). Human reads aligned to the GRCh37 reference genome by Bowtie2 (<http://bowtie-bio.sourceforge.net/bowtie2/index.shtml>) with parameters “-very-sensitive-local” were removed.

Profiles of taxonomy and metabolic pathway of each sample were respectively inferred by MetaPhlAn (v2.7.6) (<https://huttenhower.sph.harvard.edu/metaphlan2>) and HUMAnA2 (<https://huttenhower.sph.harvard.edu/humann>) with default parameters as previously described(1). For gene annotation, MetaWRAP v1.0.2 (<https://github.com/bxlab/metaWRAP>) pipeline was used for contig assembly and binning. High-quality reads were assembled using metaSPAdes (v3.12) (<https://github.com/ablab/spades>). Gene sequences were predicted using prodigal (<https://github.com/hyattpd/Prodigal>) with default parameters, and gene abundances were estimated using Salmon v0.9.1 (<https://github.com/COMBINE-lab/salmon/releases/tag/v0.9.1>) with quasi-mapping mode. The protein family sequences of carbohydrate-active enzymes and peptidases were downloaded from CAZy (http://www.cazy.org/) and MEROPS (<https://www.ebi.ac.uk/merops/>) database, respectively. All protein families were aligned to the predicted opening read frames (OFRs) using DIAMOND v0.9.10 (<https://github.com/bbuchfink/diamond/releases/tag/v0.9.10>) with 50% identity and E-value of e-10, and the abundance of each protein family in each sample was calculated by sum all ORFs abundance aligned to each family.

*Quasi-paired cohort analysis*

Quasi-paired cohort was reconstructed between SZ and healthy control (HC) samples as described previously(2). Briefly, Bray-Curtis distance metrics based on pathway profile was calculated for all SZ and HC samples. The distance to k-nearest neighbors (kNN) was inferred for each sample, where k is the square root of sample size. Samples with KNN distance > 95% or < 5% percentile are defined as “outlier samples” or “redundant samples” and removed. We then re-generated the Bray-Curtis distance metrics and identify samples inter-group KNN < intra-group KNN as “boundary samples” for both SZ and HC groups. Finally, boundary samples and their inter-group k-nearest neighbors were used to construct the quasi-paired cohort.

*Measurement of SCFAs in fecal and plasma samples*

For plasma samples, 100 μL plasma was added to a centrifuge tube, then 38 μL 50% H2SO4 and 150 μL ether (containing 0.4 μg/mL SCFAs internal standard) was added sequentially. The above solution was mixed on Vortex-5 (America) for 1 min, centrifuged at 12000 rpm for 20 min, let stand at 4 °C for 30 min. The upper ether solution was stored at -20 °C. Concentrations of SCFAs were determined using gas chromatography-mass spectrometry (GC-MS) with Agilent 7890A / 5975C GC-MS under the full scan and SIM mode. For fecal samples, 100 mg stool was transferred to grinding tube, added 800 μL water, ground at 60 Hz for 3 min, and 400 μL supernatant was collected and used to detect SCFAs.

*Measurement of medium-and long-chain free fatty acids in fecal and plasma samples*

Aliquot 300 μL plasma or 200 mg stool samples was added with 2 mL methanol and 4 mL chloroform, and mixtures were shaken at 180 rpm/min for 20 min at 25 °C. 2 mL deionized water was added to the mixture, vortexed for 2 min, and then centrifuged at 1000 rpm for 10 min to remove the upper aqueous layer. 3 mL hexane and 10 μL (10 mg/mL) C19:0 internal standard were added and vortexed for 2 min. Then 3 mL 0.4 mol/L KOH/methanol was added, vortexed for 1 min, and then incubated for 30 min at 37 °C. 2 mL deionized water was added, vortexed for 2 min, and supernatants were collected and transferred to a centrifuge tube. The sample was dried using nitrogen gas, and then 200 μL hexane was added and vortexed for 2 min, and the supernatant was transferred to an GC/MS vial. GC-MS analysis was performed on Agilent 7890A-5975C (Agilent Technologies, USA) equipped with a DB-5 capillary column (30m×0.25mm×0.25µm). The detection conditions were as follows: injection volume was 1 μL, the temperature of injection was 270 °C; the diversion ratio was 5:1; the carrier gas was helium gas (99.999%), and the flow rate was 1 mL/min; the column temperature was starting from 70 °C for 5 min, increased to 200 °C at a rate of 25 °C/min, then increased to 240 °C at a rate 2 °C /min and held for 10 min; interface temperature was 280 °C; the ion source temperature was 230 °C; quadrupole temperature 150 °C; acquisition mode was full scan. The GC/MS data peak extraction was analyzed using Agilent 5975 Data Analysis software, and metabolites were identified based on the NIST library.

*Non-targeted metabolome analysis*

For fecal samples, 50 mg stool were transferred to EP tubes containing 800 μL methanol, 5 μL ribitol (9.5 mg/mL) was added as internal standard. Samples were ground at 60 Hz for 2 min, and then sonicated in an ice bath at 4 K Hz for 30 min. 200 μL supernatant was collected after centrifugation. 30 μL methoxypyridine (20 mg/mL) was added to the supernatant, then shook vigorously for 30 s, and oximated at 37°C for 90 min. Then 30 μL BSTFA (containing 1% TMCS) was added for derivatization and reacted at 70°C for 60 min, let stand at room temperature for 30 min. Non-targeted metabolites were detected using GC-MS with Agilent 6890A / 5973C GC-MS under the full scan mode. Original data of GC-MS was imported into R and performed metabolite peak alignment, detection, and normalization using XCMS package. All features were filtered with a match score > 700, and features from the same metabolites were combined for analysis.

*Targeted detection of metabolites in stool*

Water was purified using ultrapure water preparation system; methanol and acetonitrile (LC-MS grade) were purchased from Merck (Germany); formic acid (HPLC grade ) was obtained from Sigma (Germany).

All amino acids internal standard references were purchased from Cambridge Isotope Laboratories (U.S), including L-Tryptophan-D8, L-Phenylalanine-D8, L-Proline-D7, L-Asparagine-13C4, L-Isoleucine-D10, L-Histidine-D5, L-Methionine-D3, L-Valine-D8, L-Alanine-D, L-Lysine-D9, DL-Serine-D3, L-Arginine-D7, DL-Glutamic acid-D5, L-Aspartic acid-D3, L-Glutamine-D5, Kynurenic acid-D5, Choline-D13, L-Citrulline-D4. Indole-3-acetic acid (CAS 87-51-4) was purchased from J&K. Other derivatives internal standard references were purchased from Sigma-Aldrich, including indole (120-72-9), L-DOPA (59-92-7), p-cresol (106-44-5), phenol (108-95-2), Indole-3-propionic acid (830-96-6), kynurenine (343-65-7), kynurenic acid (492-27-3) γ-aminobutyric acid (56-12-2), quinolinate (89-00-9).

The extraction method was referred to the previous paper with modification [25]. Briefly, 50 mg fecal samples were mixed with ice-cold 80% methanol in water and ground using abrader at 5000 rpm, 10 s, two cycles, pause 5 s, and incubated for 30 min at 1500 rpm and 4 oC; then centrifuge for 10 min at 12000 rpm and 4 oC. Remove the supernatant into a clean 1.5 ml centrifuge tube, and dry using SpeedVac; the dried extracts were re-dissolved with 1% acetonitrile in water, and upper layer liquids were collected for LC-MS analysis.

*Instruments for metabolome analysis*

The used instruments included hundred thousandth electronic analytical balance (Sartorius Practum, SQP, Sartorius scientific instruments (Beijing) Co., Ltd), Milli-Q Plus machine (Millipore, Brussels, Belgium); vortex mixers (Vortex-Genie2, Scientific Industries, U.S.); SpeedVac( Genevac miVac, Tegent Scientific Ltd., England); Abrader (Precellys EvolutionBertin Technologies, French).

The LC-MS conditions were referenced to the previous paper [26]. The column ACQUITY UPLC HSS T3 1.8 μm, 3.0 × 100 mm columns (Waters, Dublin, Ireland) was adopted into the present study. Ultra-performance Liquid Chromatography (Agilent 1290 II, Agilent Technologies, Germany) coupled to Quadrupole-TOF MS (5600 Triple TOF Plus, AB SCIEX, Singapore) was applied to acquire metabolome data.

The temperatures of the column and auto-sampler were controlled at 40 °C and 10 °C, respectively. The injection volume was set to 2 μL per run. The detection parameters of MS were: vaporizer temperature was 500 °C; ESI source voltages were +5.5 kV and −4.5 kV, respectively; nebulizer gas (N2) pressure was 50 psi; curtain gas (N2) pressure was 35 psi; drying gas (N2) pressure was 50 psi; The scan range was m/z 60-600.

MS/MS analyses of the metabolites were performed using information-dependent acquisition mode. The collision energy was 35 ± 15 eV. Analyst® TF 1.7.1 Software (AB Sciex, Concord, ON, Canada) was used to acquisite and process data.

*Data processing of metabolome*

All detected ions were extracted using MarkerView 1.3 (AB Sciex, Concord, ON, Canada) into Excel in the format of two dimensional matrix, including mass to charge ratio (m/z), retention time, and peak areas, and isotopic peaks were filtered. PeakView 2.2 (AB Sciex, Concord, ON, Canada) was applied to extract MS/MS data, and perform comparison with Metabolites database (AB Sciex, Concord, ON, Canada), HMDB, METLIN, and standard references to annotate ion ID. Self-compiled R program was used for statistical analysis.

**Reference:**

1. Thomas AM, Manghi P, Asnicar F, Pasolli E, Armanini F, Zolfo M, Beghini F, Manara S, Karcher N, Pozzi C, Gandini S, Serrano D, Tarallo S, Francavilla A, Gallo G, Trompetto M, Ferrero G, Mizutani S, Shiroma H, Shiba S, Shibata T, Yachida S, Yamada T, Wirbel J, Schrotz-King P, Ulrich CM, Brenner H, Arumugam M, Bork P, Zeller G, Cordero F, Dias-Neto E, Setubal JC, Tett A, Pardini B, Rescigno M, Waldron L, Naccarati A, Segata N. Metagenomic analysis of colorectal cancer datasets identifies cross-cohort microbial diagnostic signatures and a link with choline degradation. Nature medicine. 2019;25:667-678.

2. Zhang M, Chu Y, Meng Q, Ding R, Shi X, Wang Z, He Y, Zhang J, Liu J, Zhang J, Yu J, Kang Y, Wang J. A quasi-paired cohort strategy reveals the impaired detoxifying function of microbes in the gut of autistic children. Science advances. 2020;6:eaba3760.
