# Supplementary material for: Enhanced intestinal protein fermentation in schizophrenia
Source: BMC Med. 2022 Feb 9;20:67. doi: 10.1186/s12916-022-02261-z (PMC8827269; doi:10.1186/s12916-022-02261-z)
Supplement: Supplementary file 2 — Additional file 2: Figure S1. Deviated metabolites in plasma and stool samples of SZ. Figure S2. Differential medium-and long-chain free fatty acids in plasma and stool between SZ and HC. Figure S3. The diversity of gut microbiota in schizophrenia. Figure S4. Decreased carbohydrate-catabolizing activity in the intestine of SZ patients. Figure S5. Enhanced amino acid-catabolism in the intestine of SZ patients. Figure S6. The correlations of daily carbohydrate intake with the psychiatric symptoms. [file 12916_2022_2261_MOESM2_ESM.docx]

**Supplementary Figures: S1-S6**


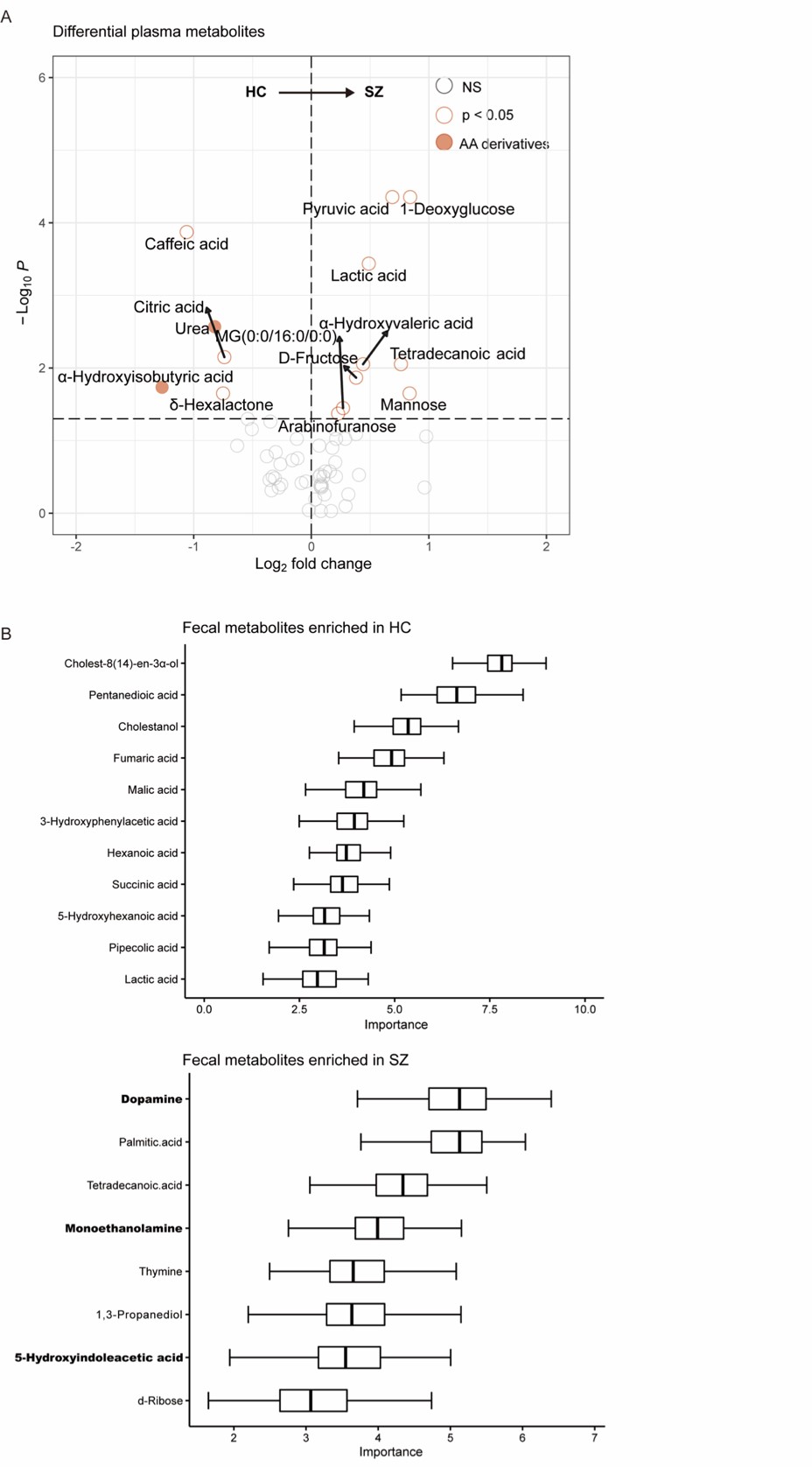


Fig. S1. Deviated metabolites in plasma and stool samples of SZ. A. Volcano plot of the differential metabolites in plasma. B. Permutation importance of metabolites in stool with highest contribution to the random forest models in favor of HC (upper panel) and SZ (lower panel).


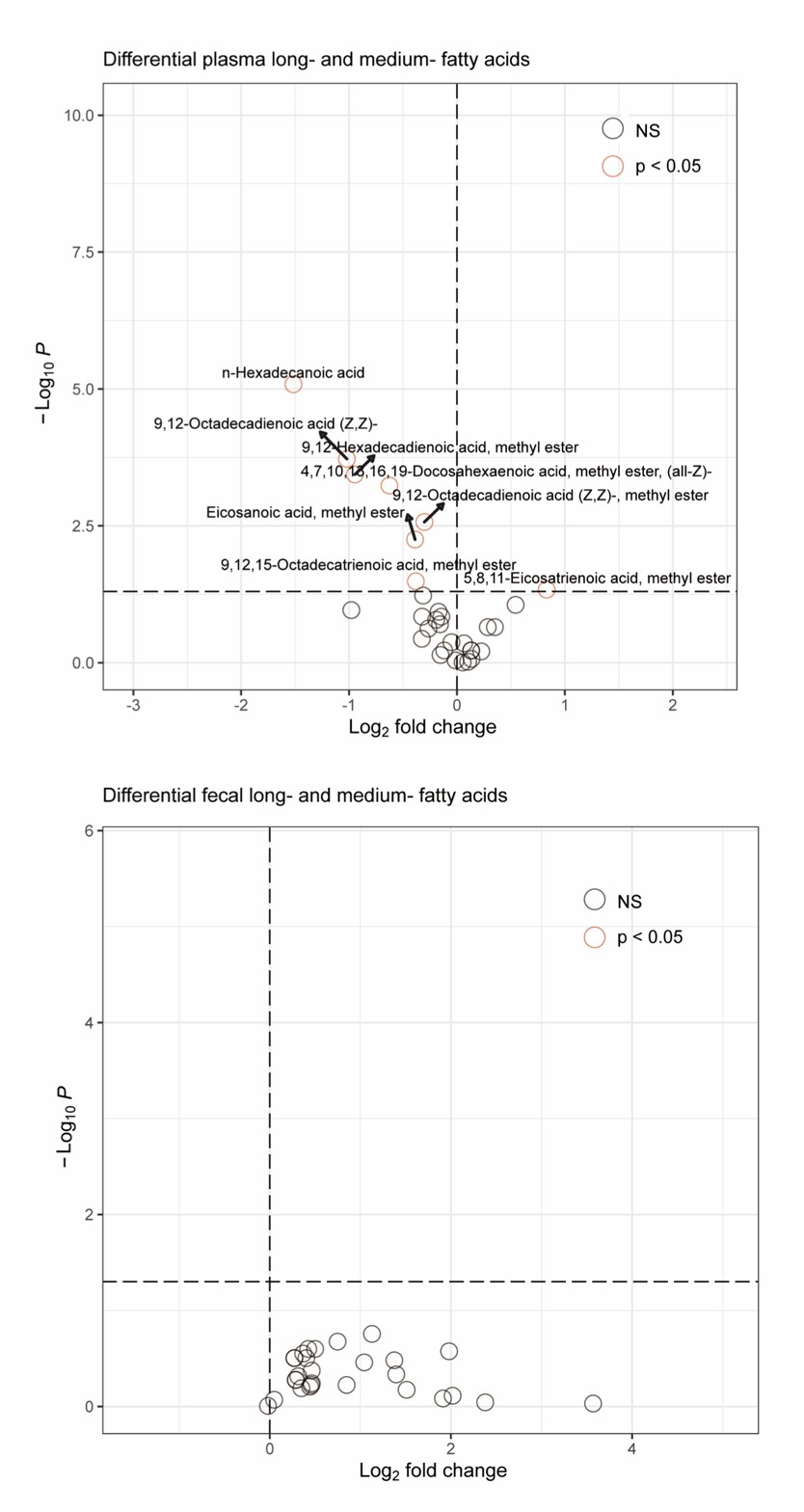


**Fig. S2. Differential medium-and long-chain free fatty acids in plasma and stool between SZ and HC.** Volcano plot of the differential fatty acids in plasma (upper panel) and (lower panel).

Fig S3. The diversity of gut microbiota in schizophrenia. (A) The Shannon index of gut microbiota in male and female SZ patients compared to controls. (B) The PCoA analysis of gut microbiota in male (left panel) and female (right panel) SZ patients compared to controls.


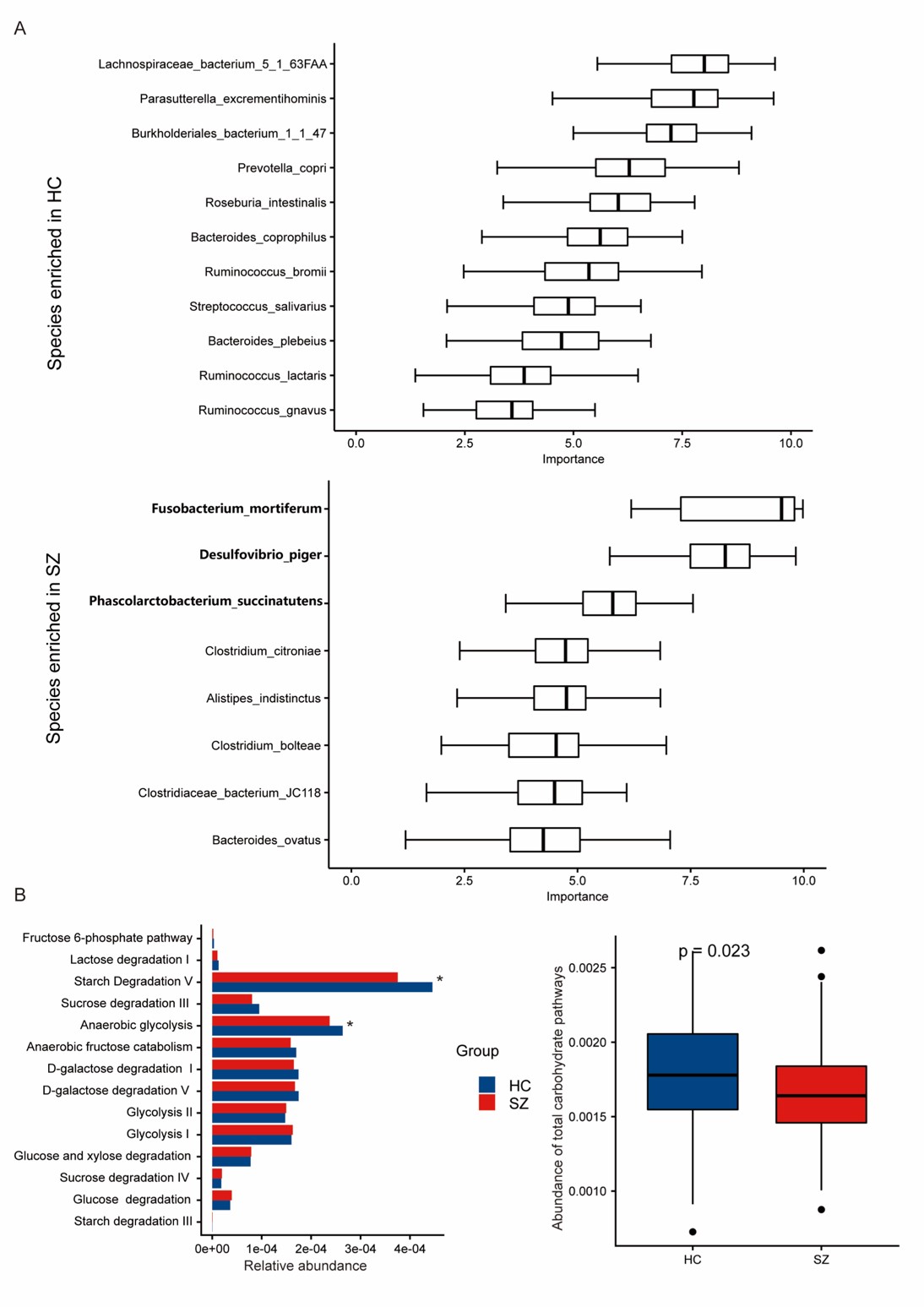


**Fig. S4. Decreased carbohydrate-catabolizing activity in the intestine of SZ patients . A.** Permutation importance of microbial species with highest contribution to the random forest models in favor of HC (upper panel) and SZ (lower panel). **B.** The relative abundance of each carbohydrate catabolism pathways (left panel) and their sum (right panel) in HC and SZ samples.


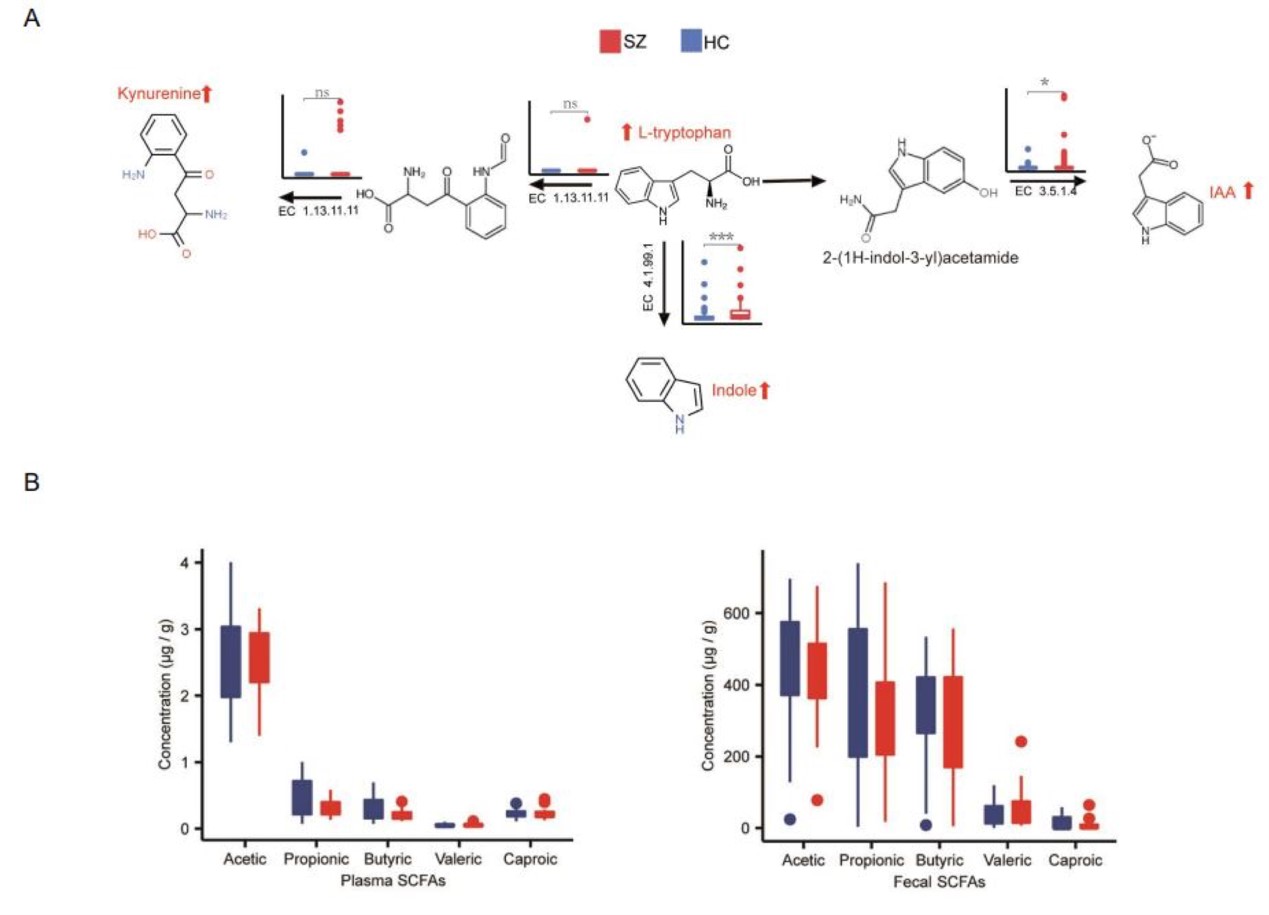


**Fig. S5. Enhanced amino acid-catabolism in the intestine of SZ patients. A.**Fecal concentrations of L-tryptophan and its derivatives kynurenine, IAA, and indole are all significantly increased in SZ when normalized by daily protein intake, and the enzymes responsible for the production of IAA (EC 3.5.1.4) and indole (EC 4.1.99.1) are also significantly elevated in SZ. IAA, indole-3-acetic acid; **B.** The concentration of short-chain fatty acids in plasma(left panel ) and fecal(right panel) between HC and SZ. HC, healthy controls; SZ, patients with schizophrenia ; *, p < 0.05; ***, p < 0.001, Wilcoxon rank-sum test.


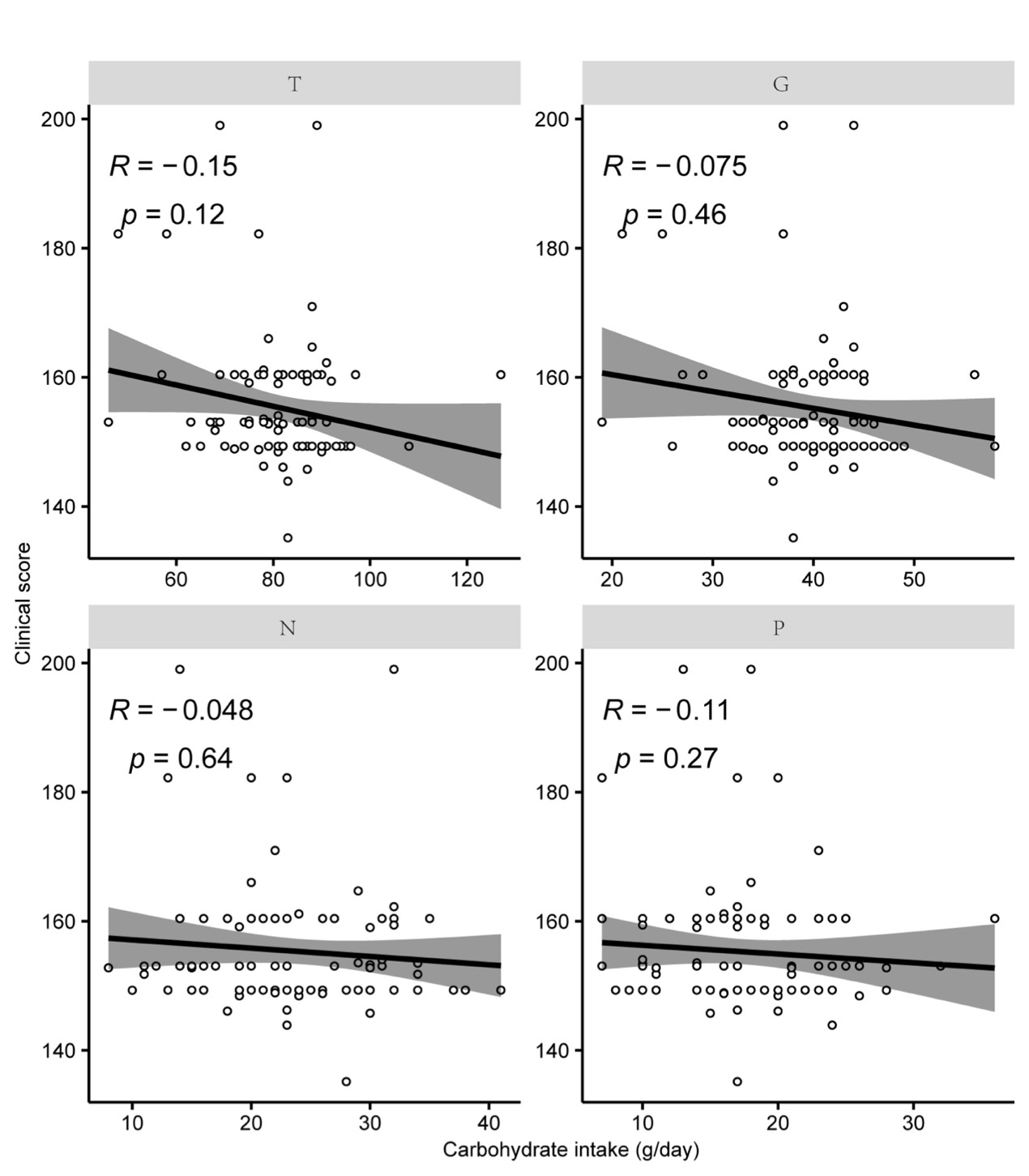


**Fig. S6. The correlations of daily carbohydrate intake with the psychiatric symptoms.**
