# Supplementary material for: Enhanced intestinal protein fermentation in schizophrenia
Source: BMC Med. 2022 Feb 9;20:67. doi: 10.1186/s12916-022-02261-z (PMC8827269; doi:10.1186/s12916-022-02261-z)
Supplement: Supplementary file 3 — Additional file 3: Table S1. Differential species in the gut microbiota between HC and SZ. Table S2. Differential metabolic pathways encoded by gut microbes between HC and SZ. [file 12916_2022_2261_MOESM3_ESM.docx]

**Supplementary Table S1-S2**

Table S1. Differential species in the gut microbiota between HC and SZ.

| **Species** | **P-value** | **Mean of SZ** | **Mean of HC** | **Fold**  **Change (SZ/HC)** | **Metabolism** |
| --- | --- | --- | --- | --- | --- |
| Clostridiaceae_bacterium_JC118 | 3.96E-04 | 0.0119 | 0.0001 | 140.15 | Uncharacterized |
| Fusobacterium_mortiferum | 3.72E-07 | 1.1630 | 0.0106 | 110.19 | Asaccharolytic |
| Lachnospiraceae_bacterium_3_1_57FAA_CT1 | 2.09E-02 | 0.2240 | 0.0166 | 13.51 | Uncharacterized |
| Desulfovibrio_piger | 6.97E-07 | 0.2775 | 0.0282 | 9.83 | Asaccharolytic |
| Clostridium_celatum | 1.59E-02 | 0.0060 | 0.0012 | 4.92 | Saccharolytic |
| Eubacterium_biforme | 1.12E-03 | 0.5316 | 0.1297 | 4.10 | Saccharolytic |
| Lachnospiraceae_bacterium_8_1_57FAA | 3.78E-02 | 0.0535 | 0.0136 | 3.94 | Uncharacterized |
| Phascolarctobacterium_succinatutens | 1.19E-04 | 4.7099 | 1.2397 | 3.80 | Asaccharolytic |
| Clostridium_asparagiforme | 6.47E-03 | 0.0289 | 0.0088 | 3.28 | Fermentative, few compounds used |
| Sutterella_wadsworthensis | 1.19E-02 | 0.2641 | 0.0849 | 3.11 | Asaccharolytic |
| Clostridium_symbiosum | 1.34E-02 | 0.0580 | 0.0201 | 2.89 | Saccharolytic |
| Alistipes_indistinctus | 5.44E-03 | 0.0895 | 0.0326 | 2.74 | Saccharolytic |
| Bacteroides_faecis | 2.77E-02 | 0.1758 | 0.0721 | 2.44 | Saccharolytic |
| Streptococcus_anginosus | 2.00E-02 | 0.0236 | 0.0121 | 1.95 | Saccharolytic |
| Odoribacter_splanchnicus | 2.96E-02 | 0.5306 | 0.2899 | 1.83 | Saccharolytic |
| Parabacteroides_merdae | 1.85E-02 | 2.6100 | 1.5057 | 1.73 | Saccharolytic |
| Clostridium_citroniae | 2.53E-03 | 0.0324 | 0.0191 | 1.70 | Saccharolytic |
| Clostridium_bartlettii | 4.85E-02 | 0.0399 | 0.0255 | 1.56 | Ferments carbohydrates and amino acids |
| Clostridium_bolteae | 3.86E-03 | 0.1122 | 0.0793 | 1.41 | Saccharolytic |
| Clostridium_nexile | 6.85E-03 | 0.5572 | 0.5423 | 1.03 | Saccharolytic |
| Prevotella_stercorea | 3.91E-02 | 1.9672 | 1.9541 | 1.01 | Saccharolytic |
| Ruminococcus_gnavus | 3.11E-03 | 0.7699 | 0.7758 | 0.99 | Saccharolytic |
| Turicibacter_sanguinis | 3.78E-02 | 0.0067 | 0.0072 | 0.93 | Uncharacterized |
| Turicibacter_unclassified | 4.65E-03 | 0.0222 | 0.0239 | 0.93 | Saccharolytic |
| Prevotella_copri | 9.37E-03 | 12.1159 | 13.5807 | 0.89 | Saccharolytic |
| Lachnospiraceae_bacterium_7_1_58FAA | 1.43E-02 | 0.0536 | 0.0617 | 0.87 | Uncharacterized |
| Ruminococcus_bromii | 1.76E-04 | 0.5130 | 0.6985 | 0.73 | Saccharolytic |
| Dorea_formicigenerans | 1.75E-02 | 0.1561 | 0.2160 | 0.72 | Saccharolytic |
| Eubacterium_hallii | 4.79E-03 | 0.0619 | 0.0987 | 0.63 | Saccharolytic |
| Roseburia_intestinalis | 9.73E-03 | 0.1569 | 0.2642 | 0.59 | Saccharolytic |
| Coprococcus_comes | 3.22E-02 | 0.1787 | 0.3382 | 0.53 | Saccharolytic |
| Parabacteroides_johnsonii | 8.29E-04 | 0.0888 | 0.1685 | 0.53 | Saccharolytic |
| Bacteroides_plebeius | 6.65E-04 | 2.5262 | 4.9616 | 0.51 | Saccharolytic |
| Bacteroides_coprocola | 1.59E-02 | 2.4090 | 5.1412 | 0.47 | Saccharolytic |
| Ruminococcus_lactaris | 9.78E-05 | 0.1330 | 0.3225 | 0.41 | Saccharolytic |
| Burkholderiales_bacterium_1_1_47 | 4.38E-06 | 0.1317 | 0.3375 | 0.39 | Uncharacterized |
| Anaerostipes_hadrus | 4.73E-03 | 0.0045 | 0.0141 | 0.32 | Saccharolytic |
| Parasutterella_excrementihominis | 3.04E-06 | 0.1143 | 0.3587 | 0.32 | Fermentative, respiratory |
| Lachnospiraceae_bacterium_5_1_63FAA | 8.98E-05 | 0.0095 | 0.0372 | 0.26 | Uncharacterized |
| Enterococcus_faecium | 2.46E-02 | 0.0011 | 0.0043 | 0.25 | Saccharolytic |
| Bacteroides_coprophilus | 2.26E-05 | 0.1811 | 1.0854 | 0.17 | Saccharolytic |
| Streptococcus_salivarius | 1.63E-04 | 0.0342 | 0.2350 | 0.15 | Saccharolytic |
| Bifidobacterium_pseudocatenulatum | 1.55E-02 | 0.0246 | 0.2265 | 0.11 | Saccharolytic |

**Table S2. Differential metabolic pathways encoded by gut microbes between HC and SZ.**

| **Metabolic pathways** | **P value** | **FDR** | **SZ Mean** | **HC Mean** | **Fold Change SZ/HC** |
| --- | --- | --- | --- | --- | --- |
| **Enriched in HC** |  |  |  |  |  |
| PWY0-1319: CDP-diacylglycerol biosynthesis II | 5.83E-07 | 1.93E-05 | 4.30E-04 | 5.09E-04 | 0.84 |
| PWY-5667: CDP-diacylglycerol biosynthesis I | 5.83E-07 | 1.93E-05 | 4.30E-04 | 5.09E-04 | 0.84 |
| PANTO-PWY: phosphopantothenate biosynthesis I | 3.24E-05 | 4.06E-04 | 4.01E-04 | 4.60E-04 | 0.87 |
| PWY-1042: glycolysis IV (plant cytosol) | 3.37E-06 | 7.43E-05 | 3.46E-04 | 4.12E-04 | 0.84 |
| PWY0-162: superpathway of pyrimidine ribonucleotides de novo biosynthesis | 5.42E-05 | 5.79E-04 | 1.84E-04 | 2.31E-04 | 0.80 |
| THRESYN-PWY: superpathway of L-threonine biosynthesis | 1.52E-08 | 1.76E-06 | 1.64E-04 | 1.89E-04 | 0.87 |
| PWY-5347: superpathway of L-methionine biosynthesis (transsulfuration) | 2.44E-07 | 9.41E-06 | 1.06E-04 | 1.20E-04 | 0.88 |
| PWY-5188: tetrapyrrole biosynthesis I (from glutamate) | 1.10E-06 | 2.83E-05 | 1.03E-04 | 1.22E-04 | 0.84 |
| PWY-5659: GDP-mannose biosynthesis | 2.16E-05 | 3.34E-04 | 8.71E-05 | 1.02E-04 | 0.86 |
| PWY-6628: superpathway of L-phenylalanine biosynthesis | 2.77E-05 | 3.80E-04 | 6.98E-05 | 7.94E-05 | 0.88 |
| HOMOSER-METSYN-PWY: L-methionine biosynthesis I | 5.27E-05 | 5.79E-04 | 6.96E-05 | 7.75E-05 | 0.90 |
| P4-PWY: superpathway of L-lysine, L-threonine and L-methionine biosynthesis I | 3.76E-09 | 6.39E-07 | 6.31E-05 | 7.46E-05 | 0.84 |
| PWY0-781: aspartate superpathway | 4.14E-09 | 6.39E-07 | 6.10E-05 | 7.34E-05 | 0.83 |
| PWY-7383: anaerobic energy metabolism (invertebrates, cytosol) | 2.96E-05 | 3.81E-04 | 5.94E-05 | 6.48E-05 | 0.92 |
| UDPNAGSYN-PWY: UDP-N-acetyl-D-glucosamine biosynthesis I | 3.82E-05 | 4.42E-04 | 5.70E-05 | 6.86E-05 | 0.83 |
| SULFATE-CYS-PWY: superpathway of sulfate assimilation and cysteine biosynthesis | 7.95E-05 | 7.76E-04 | 5.62E-05 | 6.21E-05 | 0.91 |
| PWY0-1061: superpathway of L-alanine biosynthesis | 3.75E-05 | 4.42E-04 | 5.54E-05 | 6.07E-05 | 0.91 |
| PWY-5345: superpathway of L-methionine biosynthesis (by sulfhydrylation) | 1.44E-05 | 2.38E-04 | 4.76E-05 | 5.50E-05 | 0.87 |
| P461-PWY: hexitol fermentation to lactate, formate, ethanol and acetate | 6.39E-07 | 1.97E-05 | 4.62E-05 | 6.14E-05 | 0.75 |
| NAGLIPASYN-PWY: lipid IVA biosynthesis | 4.62E-06 | 9.72E-05 | 4.34E-05 | 5.21E-05 | 0.83 |
| NAD-BIOSYNTHESIS-II: NAD salvage pathway II | 8.17E-05 | 7.76E-04 | 3.51E-05 | 4.10E-05 | 0.86 |
| HEMESYN2-PWY: heme biosynthesis II (anaerobic) | 1.81E-05 | 2.90E-04 | 3.05E-05 | 4.27E-05 | 0.71 |
| PWY-5005: biotin biosynthesis II | 2.64E-08 | 2.45E-06 | 1.93E-05 | 3.90E-05 | 0.49 |
| PWY-7237: myo-, chiro- and scillo-inositol degradation | 1.30E-06 | 3.16E-05 | 1.92E-05 | 3.39E-05 | 0.57 |
| PWY0-1277: 3-phenylpropanoate and 3-(3-hydroxyphenyl)propanoate degradation | 2.73E-05 | 3.80E-04 | 1.75E-05 | 2.60E-05 | 0.67 |
| KETOGLUCONMET-PWY: ketogluconate metabolism | 9.33E-05 | 8.47E-04 | 1.40E-05 | 1.76E-05 | 0.79 |
| HCAMHPDEG-PWY: 3-phenylpropanoate and 3-(3-hydroxyphenyl)propanoate degradation to 2-oxopent-4-enoate | 9.97E-07 | 2.72E-05 | 1.21E-05 | 2.06E-05 | 0.59 |
| PWY-6690: cinnamate and 3-hydroxycinnamate degradation to 2-oxopent-4-enoate | 9.97E-07 | 2.72E-05 | 1.21E-05 | 2.06E-05 | 0.59 |
| P185-PWY: formaldehyde assimilation III (dihydroxyacetone cycle) | 1.52E-10 | 7.03E-08 | 8.89E-06 | 1.74E-05 | 0.51 |
| 3-HYDROXYPHENYLACETATE-DEGRADATION-PWY: 4-hydroxyphenylacetate degradation | 2.34E-05 | 3.49E-04 | 7.27E-06 | 1.29E-05 | 0.56 |
| PWY-3781: aerobic respiration I (cytochrome c) | 1.11E-07 | 5.12E-06 | 3.47E-06 | 1.07E-05 | 0.33 |
|  |  |  |  |  |  |
| **Enriched in SZ** |  |  |  |  |  |
| PWY-6270: isoprene biosynthesis I | 7.14E-08 | 5.12E-06 | 1.66E-04 | 1.25E-04 | 1.32 |
| PWY-7560: methylerythritol phosphate pathway II | 7.80E-08 | 5.12E-06 | 1.54E-04 | 1.14E-04 | 1.35 |
| PWY-6168: flavin biosynthesis III (fungi) | 8.22E-06 | 1.46E-04 | 1.87E-04 | 1.63E-04 | 1.15 |
| GLCMANNANAUT-PWY: superpathway of N-acetylglucosamine, N-acetylmannosamine and N-acetylneuraminate degradation | 2.79E-05 | 3.80E-04 | 1.11E-04 | 9.95E-05 | 1.12 |
| LPSSYN-PWY: superpathway of lipopolysaccharide biosynthesis | 8.17E-05 | 7.76E-04 | 1.88E-05 | 8.48E-06 | 2.21 |
